# Reflection does not undermine self-interested prosociality

**DOI:** 10.3389/fnbeh.2014.00300

**Published:** 2014-09-03

**Authors:** David G. Rand, Gordon T. Kraft-Todd

**Affiliations:** ^1^Department of Psychology, Yale UniversityNew Haven, CT, USA; ^2^Department of Economics, Yale UniversityNew Haven, CT, USA; ^3^Organizational Behavior, School of Management, Yale UniversityNew Haven, CT, USA

**Keywords:** cooperation, economic games, prosociality, moral psychology, dual process

## Abstract

The cognitive basis of prosocial behavior has received considerable recent attention. Previous work using economic games has found that in social dilemmas, intuitive decisions are more prosocial on average. The Social Heuristics Hypothesis (SHH) explains this result by contending that strategies which are successful in daily life become automatized as intuitions. Deliberation then causes participants to adjust to the self-interested strategy in the specific setting at hand. Here we provide further evidence for the SHH by confirming several predictions regarding when and for whom time pressure/delay will and will not alter contributions in a Public Goods Game (PGG). First, we replicate and extend previous results showing that (as predicted by the SHH) trust of daily-life interaction partners and previous experience with economic games moderate the effect of time pressure/delay in social dilemmas. We then confirm a novel prediction of the SHH: that deliberation should not undermine the decision to benefit others when doing so is also individually payoff-maximizing. Our results lend further support to the SHH, and shed light on the role that deliberation plays in social dilemmas.

## Introduction

Cooperation is a key component of life, from the cells in our bodies up through our personal and professional interactions and the relationships between nations, and thus is a major focus of study across the natural and social sciences (Hardin, 1968; Ostrom, 1990; Batson and Moran, 1999; Milinski et al., 2002; Boyd et al., 2003; Fehr and Fischbacher, 2003; Bartlett and Desteno, 2006; Levin, 2006; Herrmann et al., 2008; Crockett, 2009; Cushman and Macindoe, 2009; Goetz et al., 2010; Sigmund, 2010; Zaki and Mitchell, 2011; Apicella et al., 2012; Espín et al., 2012; Piff et al., 2012; Rand and Nowak, 2013; Hauser et al., 2014; Peysakhovich et al., 2014). The individual costs of cooperation, however, pose a problem: why are people willing to help others? Here we consider this question using the dual-process model of decision-making, which posits that decisions can be thought of as resulting from competition between two general systems (Sloman, 1996; Stanovich and West, 1998; Chaiken and Trope, 1999; Miller and Cohen, 2001; Kahneman, 2003; Frederick, 2005): one that is fast, automatic, and intuitive; and another that is slow, controlled, and deliberative.

Using this dual-process perspective to consider prosociality, the following questions arise (Zaki and Mitchell, 2013): are we intuitively selfish and only cooperate through active self-control? Or is our automatic predisposition to be cooperative, with deliberation favoring selfishness? To shed light on this issue, recent studies have examined the effect of experimentally manipulating the level of intuition vs. deliberation on prosociality in economic games. Doing so using time pressure/delay (Rand et al., 2012, 2014a,b), cognitive load (Cornelissen et al., 2011; Schulz et al., 2014), or application of transcranial direct current stimulation to the right lateral prefrontal cortex (Ruff et al., 2013) has suggested that deliberation favors selfishness. Other studies have found no significant effect of cognitive load (Hauge et al., 2009) or time pressure (Tinghög et al., 2013; Verkoeijen and Bouwmeester, 2014), but no studies to our knowledge find a significant positive effect of deliberation on prosociality in economic games. (Some studies have used decision time *correlations* to try to gain insight into the role of intuition vs. deliberation and find opposing results Rubinstein, 2007; Piovesan and Wengström, 2009; Rand et al., 2012; Nielsen et al., 2014; recent work, however, explains these inconsistencies by demonstrating that fast response times are not a good proxy for intuitive decision-making, and that actual cognitive process *manipulations* are required instead of just correlational analyses Evans et al., 2014).

To explain this overall negative effect of deliberation on cooperation, we have proposed the “Social Heuristics Hypothesis” (SHH) (Rand et al., 2014b). The SHH adds a dual process perspective to previous theories related to cultural differences and norm internalization (Bowles and Gintis, 2002, 2003; Henrich et al., 2005, 2010; Chudek and Henrich, 2011). Specifically, the SHH posits that people adopt strategies that are successful in daily life as default (automatically applied) heuristics for social interaction. In new or atypical social situations, one's first response is to apply these heuristics. Deliberation then tailors responses to the details of the present situation. Based on this logic, the SHH makes specific predictions about when deliberation should and should not undermine one-shot anonymous cooperation. In this paper, we test three such predictions by examining cooperation in a one-shot Public Goods Game (PGG) where decisions are made under time pressure (i.e., made more intuitive) or time delay (i.e., made more deliberative).

First, in a standard one-shot anonymous social dilemma, intuition should favor the behavior which is typically payoff-maximizing in one's lives daily life, while deliberation should always favor selfishness (because selfishness is payoff maximizing in one-shot anonymous social dilemmas). The presence in daily life of repeated interactions, reputation, and the threat of sanctions typically makes cooperation payoff-maximizing outside the lab: if others will only cooperate with you when you have behaved cooperatively in the past, self-interest dictates that you cooperate; and as a result, most people choose to cooperate under these circumstances (Axelrod, 1984; Milinski et al., 2002; Dal Bó, 2005; Nowak and Sigmund, 2005; Rand et al., 2009; Dal Bó and Fréchette, 2011; Fudenberg et al., 2012; Rand and Nowak, 2013). We argue that this is the case for most subjects in lab experiments, who live in Western communities with strong institutions and norms of cooperation. Therefore, we expect that most subjects will have high levels of inter-personal trust, and as a result peoples' intuitions will favor cooperation on average.

But this should not be true for everyone: even in contexts where reciprocity is possible, if most of the people you interact with are defectors, then you maximize your payoff by also defecting (leading to the formation of non-cooperative intuitions). Therefore, promoting deliberation should only undermine cooperation among people whose daily life interaction partners are cooperative (and thus have developed cooperative intuitions). People who live in a world where most others are non-cooperative have defection as their default, and thus should be unaffected by cognitive process manipulations in the context of one-shot economic games.

Preliminary support for this prediction comes from the correlational results of Rand et al. (2012)'s Study 10, where faster decisions were more cooperative among people with high interpersonal trust, but decision time did not predict cooperation among those with low interpersonal trust. (Furthermore, the median level of trust in Rand et al. (2012)'s Study 10 was 7 on a 10 point scale, and nearly twice as many subjects were above the scale mid-point as compared to below, supporting our suggestion that most subjects are trusting). This prediction is also supported by work showing that exposure to laboratory environments where cooperation was either advantageous (long repeated Prisoner's Dilemma games) or disadvantageous (short repeated Prisoner's Dilemmas) influenced subsequent behavior in one-shot anonymous games among subjects who relied on heuristics, but not among those who were deliberative (Peysakhovich and Rand, 2013). Here we seek to test this prediction using an actual experimental manipulation of cognitive process, rather than just decision-time correlations or individual differences in cognitive style.

Second, at the heart of the SHH is overgeneralization: intuitive responses from daily life get misapplied in the one-shot anonymous interactions of the laboratory. Thus, we would not expect intuitions to favor cooperation among subjects that have substantial previous experience with one-shot economic game experiments, as they will have had an opportunity to recalibrate their automatic responses (or to learn to be on guard against them). Prior support for this prediction comes from Rand et al. (2012)'s Study 9, in which a writing exercise that induced an intuitive mindset resulted in more cooperation than one inducing a deliberative mindset, but only among subjects that were inexperienced with economic game experiments (i.e., “naïve”). Additional support comes from Rand et al. (2014b), where (i) cooperation under time pressure in experiments run on MTurk systematically decreased over a 2 year period, during which time the MTurk subject pool became much more experienced with behavioral experiments, and (ii) this pattern was reproduced in a single experiment where cooperation was higher under time pressure than time delay among naïve subjects, but did not differ based on time constraint among experienced subjects.

There remains some question regarding the role of naivety, however, as Verkoeijen and Bouwmeester (2014) found no effect of time pressure/delay in a sample of subjects all reporting to be naïve (note, however, that in this study naivety was assessed at the outset of the experiment, and it was made clear that only naïve subjects would be allowed to participate; thus there is reason to believe that many subjects may have under-reported their level of experience). Here, we thus seek to again replicate the moderating effect of naivety, and to test for the joint moderation of naivety and interpersonal trust (i.e., intuition is only predicted to favor cooperation among subjects who are both naïve and trusting).

Third, deliberation should *not* reduce prosociality in settings where no conflict exists between the individual and group (such as the games used in Saijo and Nakamura, 1995; Brandts et al., 2004; Kummerli et al., 2010). If, because of a modified payoff-structure, the collectively optimal action is also the individually optimal action, deliberative decisions should be just as prosocial as intuitive decisions. Therefore, we predict that the presence of a social dilemma should moderate the effect of deliberation on cooperation. This is not a trivial prediction: if deliberation was reducing contributions in previous experiments for a reason other than the pursuit of self-interest, for example a desire to avoid extreme responses, then it should continue to do so even with this altered payoff structure. Here we present the first test of this prediction.

## Experimental design

To assess these three predictions, we recruited 963 American participants (38% female, mean age 30.9 years) using the online labor market Amazon Mechanical Turk (MTurk; Paolacci et al., 2010; Buhrmester et al., 2011; Horton et al., 2011; Amir et al., 2012; Rand, 2012) to play a single one-shot PGG in groups of four. In keeping with standard wages on MTurk, each participant received a $0.50 show-up fee, and then chose how much of a 40 cent endowment to keep vs. contribute to a “common project” (using a radio button with options of 0, 10, 20, 30, and 40 cents, and having no default selected). All contributions were multiplied by a factor *x* and split evenly among the four group members. Subjects made their decisions asynchronously, and payoffs were determined using *ex post* matching. No deception was used, and this research was approved by the Yale University Human Subjects Committee.

To manipulate the relative role of intuition vs. deliberation, a time constraint was imposed on the decision screen. In the “Time Pressure” condition, participants were asked to decide as quickly as possible and given at most 10 s (a timer counted down from 10). In the “Time Delay” condition, participants were asked to carefully consider their decision and told to wait at least 10 s before deciding (a timer counted up from 0). Participants were only notified about the time constraint upon arriving at the screen where they had to make their contribution decision, to prevent them from deliberating ahead of time to a greater extent in the time pressure condition (Rand et al., 2013). A total of 7.2% of participants did not obey the time constraint. We include these subjects in our analysis to avoid selection problems that impair causal inference, as highlighted by Tinghög et al. (2013).

To evaluate our first two predictions, we set *x* = 2, creating a social dilemma: the aggregate payoff of all group members (i.e., social welfare) is maximized by contributing everything, but each individual receives only 1 cent back for every 2 cents contributed and thus loses money on contributing. In a post-experimental questionnaire, we followed Rand et al. (2012) and assessed the cooperativeness of participants' daily life interaction partners by asking “To what extent do you feel you can trust other people that you interact with in your daily life?” using a 7 point scale from “Very little” to “Very much” (mean 4.69, median 5; 16.8% below mid-point; 61.3% above mid-point). We also assessed whether participants had previous experience with economic games by asking “To what extent have you participated in other studies involving the dividing up of money on MTurk before taking this HIT?” using a 5 point scale from “Never” to “Very often.” We follow Rand et al. (2012, 2014b) and categorize participants as Naïve if they answered “Never” (15% of participants were Naïve). Combining predictions 1 and 2 we predict a positive three-way interaction between time pressure, trust, and naivety, such that increasing intuitiveness increases cooperation only among subjects who are both trusting and naïve.

We test our third prediction by setting *x* = 6. Here, the social dilemma disappears: for each unit a player contributes, she receives 1.5 units back from the pool, so all players contributing everything is both socially optimal and individually optimal. Thus, if deliberation undermines cooperation in social dilemmas because of a focus on self-interest, we should find no effect of manipulating deliberation in this “No Dilemma” condition. This leads us to predict no effect of time pressure in the No Dilemma condition, and a positive four-way interaction between time pressure, trust, naivety, and being in the Social Dilemma condition.

After making their decision, participants were asked which contribution amount maximized the group's payoff ($0.40 in both Dilemma and No Dilemma conditions), and which amount maximized their individual payoff ($0.00 in Dilemma, $0.40 in No Dilemma). Comprehension is assessed after the decision rather than beforehand to avoid inducing a deliberative mindset, as per (Rand et al., 2012). A total of 31.6% of subjects answered one or both questions incorrectly (this rate of non-comprehension is well in line with previous studies using economic games on Mechanical Turk, Horton et al., 2011; Rand et al., 2012, 2014b; Engel and Rand, 2014). As our central manipulation was the alteration of the payoff structure to remove the social dilemma in the No Dilemma condition, we exclude subjects who failed the comprehension questions in our main analyses. Comparing the Social Dilemma and No Dilemma conditions, the fraction of subjects incorrectly answering the question about the socially optimal choice did not vary significantly [Pearson chi2_(1)_ = 0.10, *p* = 0.76], but significantly more subjects in the No Dilemma condition gave the incorrect answer for the individually optimal choice [20.0% in Social Dilemma, 41.1% in No Dilemma, Pearson chi2_(1)_ = 52.9, *p* < 0.001]. To address potential selection bias concerns when comparing the Social Dilemma and No Dilemma conditions, we replicate our cross-condition analyses including non-comprehenders and show that the results are qualitatively equivalent.

Our analyses were performed using linear regression with robust standard errors, taking contribution amount as the dependent variable.

## Results

**Figure 1:**
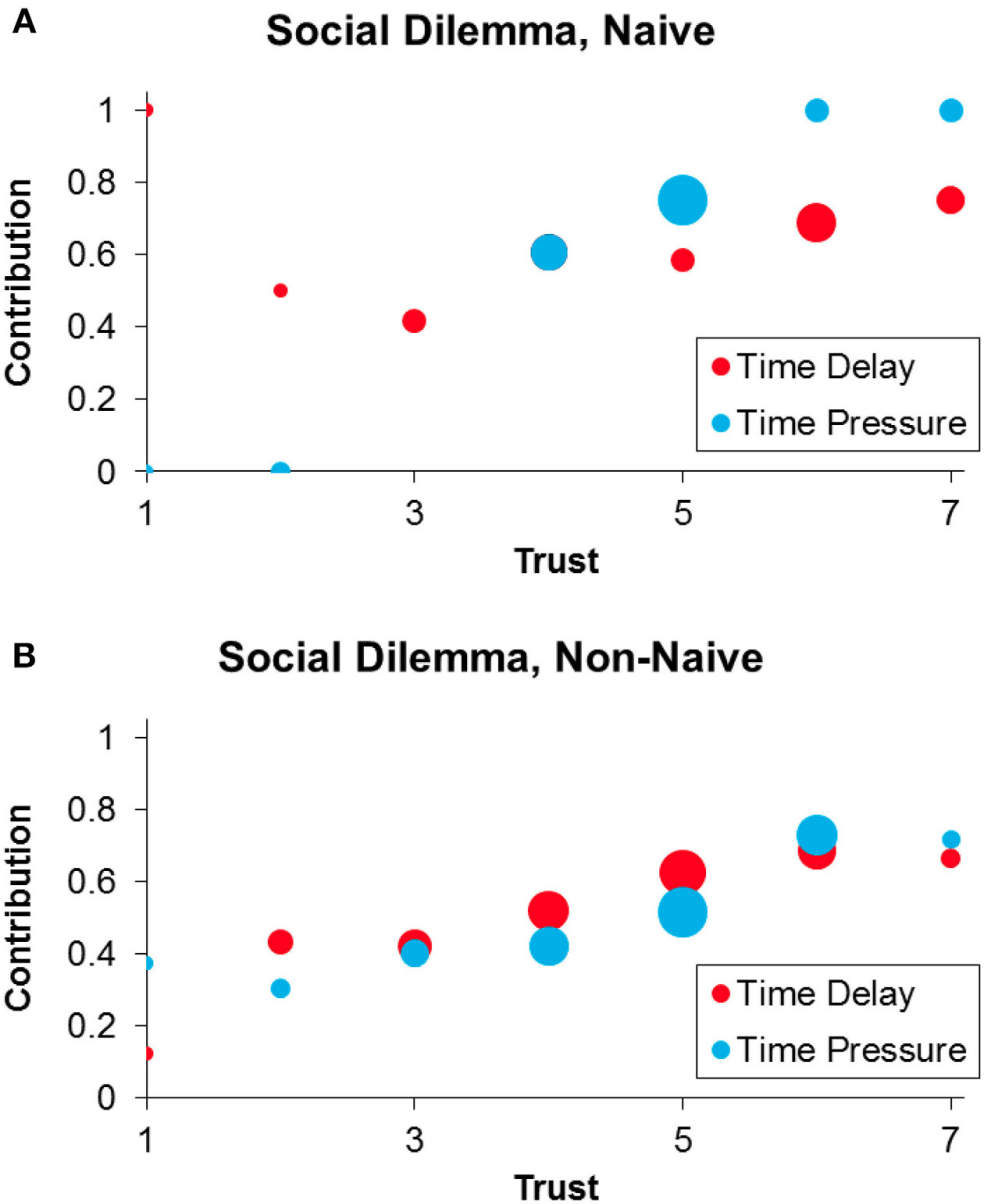
**Contributions in the Social Dilemma (*x* = 2) condition under time delay (red) and time pressure (blue), among naïve (A) and non-naïve (B) participants**. Within each panel, dot sizes are proportional to number of observations.

We begin by examining the Social Dilemma condition (Figure 1, *x* = 2) and evaluating our first two predictions regarding the joint moderation of time pressure by naivety and trust. We find the predicted positive three-way interaction between time pressure, naivety and trust when predicting contribution (Table 1 Col 2, *p* = 0.013; including demographic controls: Table 1 Col 3, *p* = 0.012): among naïve subjects that are high in trust, time pressure increases contribution. Moreover, when restricting to subjects that are both naïve and have above median trust (the group the SHH predicts should have cooperative intuitions), time pressure significantly increases contribution relative to time delay (coeff = 11.7, *p* = 0.023). Thus, we confirm our first two predictions.

**Table 1:** **Linear regressions with robust standard errors predicting PGG contribution in the Social Dilemma condition**.

	**Social dilemma (*x* = 2)**		
	**(1)**	**(2)**	**(3)**
Time pressure (TP)	−1.453 (1.655)	−4.666 (5.970)	−4.594 (6.027)
Naïve	4.018 (2.217)	11.46 (10.33)	12.16 (10.65)
Trust	3.401^**^ (0.570)	3.059^**^ (0.891)	2.982^**^ (0.886)
TP × Naïve		−24.17 (12.44)	−24.86^*^ (12.59)
TP × Trust		0.570 (1.257)	0.554 (1.260)
Naïve × Trust		−1.988 (2.022)	−2.020 (2.088)
**TP × Naïve** × **Trust**		5.994^*^ (2.390)	6.131^*^ (2.435)
Age			0.172^*^ (0.0804)
Female			−0.960 (1.731)
Education dummies	No	No	Yes
Constant	7.063^*^ (2.790)	8.897^*^ (4.097)	27.68^**^ (3.300)
Observations	395	395	395
R-squared	0.088	0.102	0.125

*Subjects failing the comprehension questions are excluded*.

*Robust standard errors in parentheses*.

** *p < 0.01*,

* *p < 0.05*.

**Figure 2:**
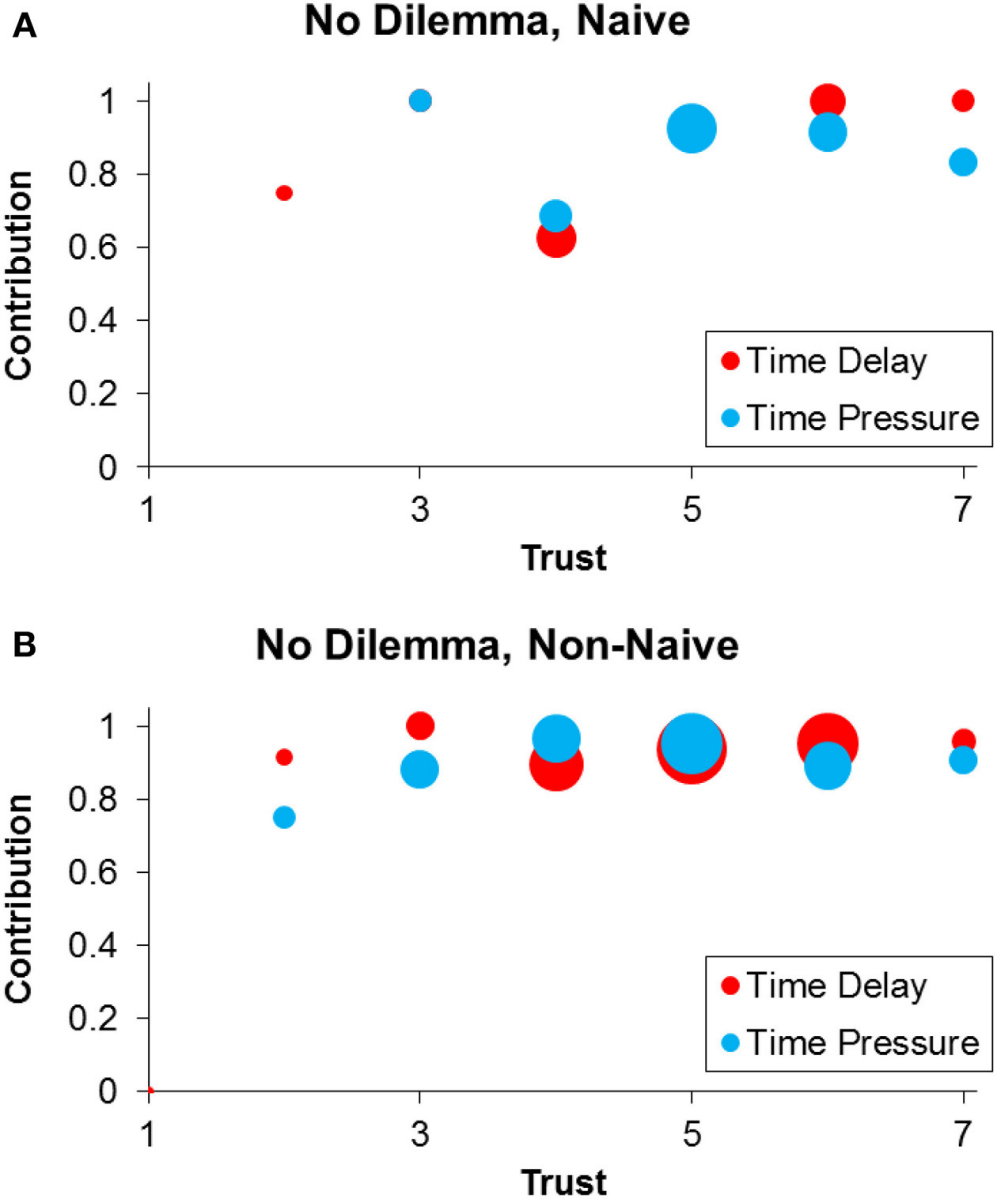
**Contributions in the No Dilemma (*x* = 6) condition under time delay (red) and time pressure (blue), among naïve (A) and non-naïve (B) participants**. Within each panel, dot sizes are proportional to number of observations.

Next we evaluate our third prediction by examining the effect of time pressure on contribution in the No Dilemma condition (Figure 2). As predicted, we find no significant main effect of time pressure (Table 2 Col 1, *p* = 0.93), and no significant interactions involving time pressure (Table 2 Col 3 and 4, all *p* > 0.40).

**Table 2:** **Linear regressions with robust standard errors predicting PGG contribution in the No Dilemma condition**.

	**No dilemma (*x* = 6)**		
	**(1)**	**(2)**	**(3)**
Time pressure (TP)	−0.0847 (0.998)	3.632 (6.163)	2.898 (6.308)
Naïve	−2.305 (1.598)	−10.23 (10.13)	−12.83 (10.50)
Trust	0.925 (0.533)	1.186 (0.905)	0.771 (0.982)
TP × Naïve		8.817 (14.51)	9.877 (14.59)
TP × Trust		−0.791 (1.205)	−0.601 (1.227)
Naïve × Trust		1.598 (1.670)	2.228 (1.753)
TP × Naïve × Trust		−1.680 (2.543)	−1.958 (2.570)
Age			0.0865^**^ (0.0316)
Female			1.055 (0.879)
Education dummies	No	No	Yes
Constant	32.62^**^ (2.879)	31.37^**^ (4.769)	34.32^**^ (3.751)
Observations	292	292	292
R-squared	0.027	0.036	0.065

*Subjects failing the comprehension questions are excluded*.

*Robust standard errors in parentheses*.

** *p < 0.01*.

To support the meaningfulness of this null result, we conduct a power calculation based on the meta-analysis of Rand et al. (2014b) where the average effect of time pressure in PGGs was found to be an increase in contribution of 7.22% of the endowment. Our sample size of 292 comprehending subjects in the No Dilemma condition is large enough to detect an effect of that size with power of 0.84. Therefore, it is unlikely that we failed to find a significant effect due to lack of power.

Furthermore, our central prediction was not this null result, but instead a predicted positive four-way interaction between time pressure, naivety, trust and a Social Dilemma dummy when combining data from both conditions. Indeed, we find this four-way interaction to do be significant (Table 3 Col 1, *p* = 0.028; including demographic controls: Table 3 Col 2, *p* = 0.028). Furthermore, when restricting to subjects that are both naïve and have a higher-than-median level of trust, we find a significant positive interaction between time pressure and the Social Dilemma condition (*p* = 0.005). Thus, we confirm our third prediction.

**Table 3:** **Linear regressions with robust standard errors predicting PGG contribution across both conditions**.

	**(1)**	**(2)**	**(3)**	**(4)**
Time pressure (TP)	3.632 (6.150)	4.126 (6.237)	−4.467 (5.457)	−4.827 (5.457)
Naïve	−10.23 (10.11)	−12.99 (10.42)	−24.27^**^ (8.647)	−25.29^**^ (8.541)
Trust	1.186 (0.903)	0.936 (0.961)	0.283 (0.842)	0.0926 (0.867)
Social dilemma (SD)	−22.47^**^ (6.284)	−22.89^**^ (6.403)	−26.33^**^ (5.761)	−26.57^**^ (5.798)
TP × Naïve	8.817 (14.48)	9.129 (14.69)	22.35^*^ (10.81)	21.53^*^ (10.97)
TP × Dilemma	−8.299 (8.578)	−8.958 (8.608)	1.401 (7.783)	1.463 (7.773)
TP × Trust	−0.791 (1.202)	−0.807 (1.216)	0.924 (1.100)	1.014 (1.099)
Naïve × SD	21.69 (14.46)	24.38 (14.72)	37.44^**^ (13.21)	38.76^**^ (13.31)
Naïve × Trust	1.598 (1.666)	2.305 (1.732)	4.289^**^ (1.532)	4.577^**^ (1.516)
Trust × SD	1.873 (1.269)	2.029 (1.295)	2.846^*^ (1.169)	2.907^*^ (1.176)
TP × Naïve × SD	−32.99 (19.10)	−33.24 (19.26)	−37.04^*^ (17.18)	−36.40^*^ (17.43)
TP × Naïve × Trust	−1.680 (2.537)	−1.781 (2.577)	−3.915^*^ (1.985)	−3.748 (2.019)
TP × Trust × SD	1.360 (1.741)	1.414 (1.744)	−0.943 (1.593)	−0.944 (1.590)
Naïve × Trust × SD	−3.585 (2.623)	−4.172 (2.666)	−6.857^**^ (2.526)	−7.143^**^ (2.557)
**TP × Naïve × Trust × SD**	7.673^*^ (3.489)	7.734^*^ (3.522)	7.957^*^ (3.374)	7.803^*^ (3.430)
Failed Comprehension			−7.813^**^ (1.107)	−7.676^**^ (1.116)
Failed Comprehension × SD			11.50^**^ (2.133)	11.19^**^ (2.160)
Age		0.139^**^ (0.0467)		0.0871^*^ (0.0433)
Female		−0.112 (1.071)		0.0900 (0.917)
Education dummies	No	Yes	No	Yes
Constant	31.37^**^ (4.759)	41.94^**^ (8.509)	35.61^**^ (4.305)	37.83^**^ (7.792)
Observations	687	687	963	962
R-squared	0.276	0.291	0.214	0.223

*Subjects failing the comprehension questions are excluded in columns 1 and 2, and included in columns 3 and 4*.

*Robust standard errors in parentheses*.

** *p < 0.01*,

* *p < 0.05*.

We now address potential concerns related to selection effects arising from the fact that more subjects failed the comprehension question regarding individually optimal behavior (and thus were excluded) in the No Dilemma condition. To do so, we include all subjects regardless of whether they failed the comprehension questions, and add a control for failing comprehension. We also include an interaction between the Social Dilemma dummy and the failed comprehension dummy, because in the Social Dilemma condition, comprehension failure (i.e., not understanding it is a dilemma) would be expected to increase giving, whereas comprehension failure in the No Dilemma condition (i.e., not understanding that there is no dilemma) would be expected to decrease giving. Doing so, we continue to find a significant four-way interaction between time pressure, naivety, trust and being in the Social Dilemma condition (Table 3 Col 3, *p* = 0.019; with demographic controls: Table 3 Col 4, *p* = 0.023). Thus, the difference we showed above in the effect of time pressure between the Social Dilemma and No Dilemma conditions is not the result of excluding non-comprehenders.

Finally, we note that there is no evidence of a potentially confounding relationship between trust and naivety: trust levels do not differ significantly between naïve and experienced subjects [*t*-test, *t*_(960)_ = −0.05, *p* = 0.96].

## Discussion

Here we have examined the effect of aligning individual and group incentives on intuitive (time pressured) and reflective (time delayed) public goods provisioning. We extended earlier results (Rand et al., 2012, 2014b) by showing that time pressure increased cooperation in a social dilemma only among participants who were both naïve and trusting. We then showed that promoting intuition had no effect on cooperation when the conflict between individual and collective incentives was removed (by making contribution individually payoff maximizing). These results confirm our predictions generated by the SHH (Rand et al., 2014b), and provide evidence that deliberation undermines cooperation in social dilemmas specifically by leading participants toward self-interest.

An important limitation of the current study is that our assessment of the moderating roles of trust and prior experience takes an individual differences approach, rather than an experimental approach. Both of these measures are self-reports, and thus may be prone to inaccuracy. Moreover, the trust measure used here (and in Rand et al., 2012) is a generalized trust measure, which may be less effective at tapping into trust in interpersonal interactions than other more targeted measures (Simpson, 2007). One might also worry that choosing to cooperate in the PGG *makes* people report a high level of trust in the subsequent demographics questionnaire. However, our data suggest that this is not so: if this was the case, we should expect a main effect of trust on cooperation and not the interaction that we observe. The three-way interaction we find between trust, experience, and condition (Prediction 1) provides some evidence that time pressure makes people more cooperative when they are trusting and inexperienced, rather than trust making people more cooperative overall. Further, subjects learned the outcome of the group decision *after* making trust judgments. Therefore, it is not possible that the behavior of the group affected trust judgments. Nonetheless, future work should examine the effect of directly manipulating trust and experience on cooperation under time pressure/delay.

Another important limitation involves our study's sample size. Although we recruited a large number of subjects (*n* = 963), our four-way interaction structure (payoff structure × time constraint × trust of daily life interaction partners × naivety) and high rate of comprehension failure meant that we wound up with relatively few subjects in each bin. In particular, we had only 34 subjects who were naïve, had higher than median trust, and passed the comprehension checks. Thus, future studies are needed, using even larger sample sizes, to assess the robustness of our findings.

The SHH predicts that prior experience with economic games will reduce the effect of time pressure in the social dilemma (Rand et al., 2012, 2014b). The mechanism by which this occurs, however, remains somewhat unclear. There are two possibilities. One is that with sufficient experience, subjects develop new default responses tailored to one-shot anonymous games. Alternatively, it could be that experience with economic game experiments (and psychological experiments more generally) does not change subjects' default responses, but instead teaches them not to rely on those defaults; repeatedly exposing subjects to situations in which their defaults lead them astray may undermine their faith in the accuracy of their intuitions. The present study helps to differentiate between these possibilities in two different ways.

First, the No Dilemma condition lets us look for evidence of remodeled intuitions. If subjects developed new non-cooperative defaults for one-shot economic games (where it is typically payoff maximizing to not contribute), we might expect time pressure to reduce cooperation among experienced subjects in the No Dilemma condition: remodeled intuitions would favor non-contribution while deliberation would cause people to realize that contributing was payoff-maximizing in the variant. Yet we find no significant effect of time pressure among experienced subjects in the No Dilemma condition (coeff = −0.34, *p* = 0.737). Thus, it seems our subjects have not developed new non-cooperative intuitions.

Second, we do find evidence that experienced subjects are more skeptical of their intuitive responses. As an exploratory measure, our post-experimental questionnaire included one item from the “Faith in intuition” scale (Epstein et al., 1996) which asks how much subjects agree with the statement “I trust my initial feelings about people” using a 5 point Likert scale from “Very untrue” to “Very true.” This particular item was selected because Epstein et al. (1996) found it to be the item that loaded most heavily on their “faith in intuition” factor. We find that among those passing the comprehension checks, naïve subjects report significantly higher agreement (Mean 3.893, SE 0.085) compared to experienced subjects [Mean 3.700, SE 0.035; *t*-test *t*_(685)_ = 2.12, *p* = 0.034]. In particular, naïve subjects are significantly more likely to report maximum agreement [“Very true”; naïve 24.3%, experienced 14.4%; chi2_(1)_ = 6.41, *p* = 0.011]. Although the magnitudes of these differences are not so large, they provide preliminary evidence that experience with experiments undermines subjects' faith in their intuition, rather than remodeling the contents of those intuitions.

Based on the SHH, one might expect that in the No Dilemma condition, time pressure would *decrease* cooperation in low-trust subjects (because their intuitions should favor selfishness, while deliberation makes them realize that here it is advantageous to contribute). While we did not observe such an interaction, this is likely the result of having very few truly low-trust subjects in our sample (only 16.8% of subjects reported trust levels below the mid-point of the scale). Thus, we did not have sufficient power to detect such an effect. Examining this possibility is an important direction for future work, perhaps using cross-cultural studies in cultures with overall lower trust.

In addition to illuminating the cognitive underpinnings of cooperation, our findings may have important implications for policies aimed at increasing contributions to the public good. They suggest that in situations where people believe that it is individually costly for them to contribute, deliberation may undermine cooperation. However, when it is clear that contribution is good for the individual as well as for the group, cooperation is safe from negative effects of deliberation. Therefore, wherever organization structures are aimed at aligning individual and collective interests, such as reputation systems or profit sharing, this alignment should be made salient. Not only could this increase overall cooperation, but it could in particular facilitate cooperation in the contexts that rely on rational, deliberative decision-making.

### Conflict of interest statement

The authors declare that the research was conducted in the absence of any commercial or financial relationships that could be construed as a potential conflict of interest.
